# Motion sickness: more than nausea and vomiting

**DOI:** 10.1007/s00221-014-4008-8

**Published:** 2014-06-25

**Authors:** James R. Lackner

**Affiliations:** 1 Volen Center for Complex Systems, Brandeis University, Waltham, MA 02454 USA; 2 Ashton Graybiel Spatial Orientation Laboratory, MS 033, Brandeis University, Waltham, MA 02454 USA

**Keywords:** Motion sickness, Sopite syndrome, Adaptation, Vestibular function, Weightlessness, Visceral afferents

## Abstract

Motion sickness is a complex syndrome that includes many features besides nausea and vomiting. This review describes some of these factors and points out that under normal circumstances, many cases of motion sickness go unrecognized. Motion sickness can occur during exposure to physical motion, visual motion, and virtual motion, and only those without a functioning vestibular system are fully immune. The range of vulnerability in the normal population varies about 10,000 to 1. Sleep deprivation can also enhance susceptibility. Systematic studies conducted in parabolic flight have identified velocity storage of semicircular canal signals—velocity integration—as being a key factor in both space motion sickness and terrestrial motion sickness. Adaptation procedures that have been developed to increase resistance to motion sickness reduce this time constant. A fully adequate theory of motion sickness is not presently available. Limitations of two popular theories, the evolutionary and the ecological, are described. A sensory conflict theory can explain many but not all aspects of motion sickness elicitation. However, extending the theory to include conflicts related to visceral afferent feedback elicited by voluntary and passive body motion greatly expands its explanatory range. Future goals should include determining why some conflicts are provocative and others are not but instead lead to perceptual reinterpretations of ongoing body motion. The contribution of visceral afferents in relation to vestibular and cerebellar signals in evoking sickness also deserves further exploration. Substantial progress is being made in identifying the physiological mechanisms underlying the evocation of nausea, vomiting, and anxiety, and a comprehensive understanding of motion sickness may soon be attainable. Adequate anti-motion sickness drugs without adverse side effects are not yet available.

## Motion sickness is a complex syndrome

**Fig. 1:**
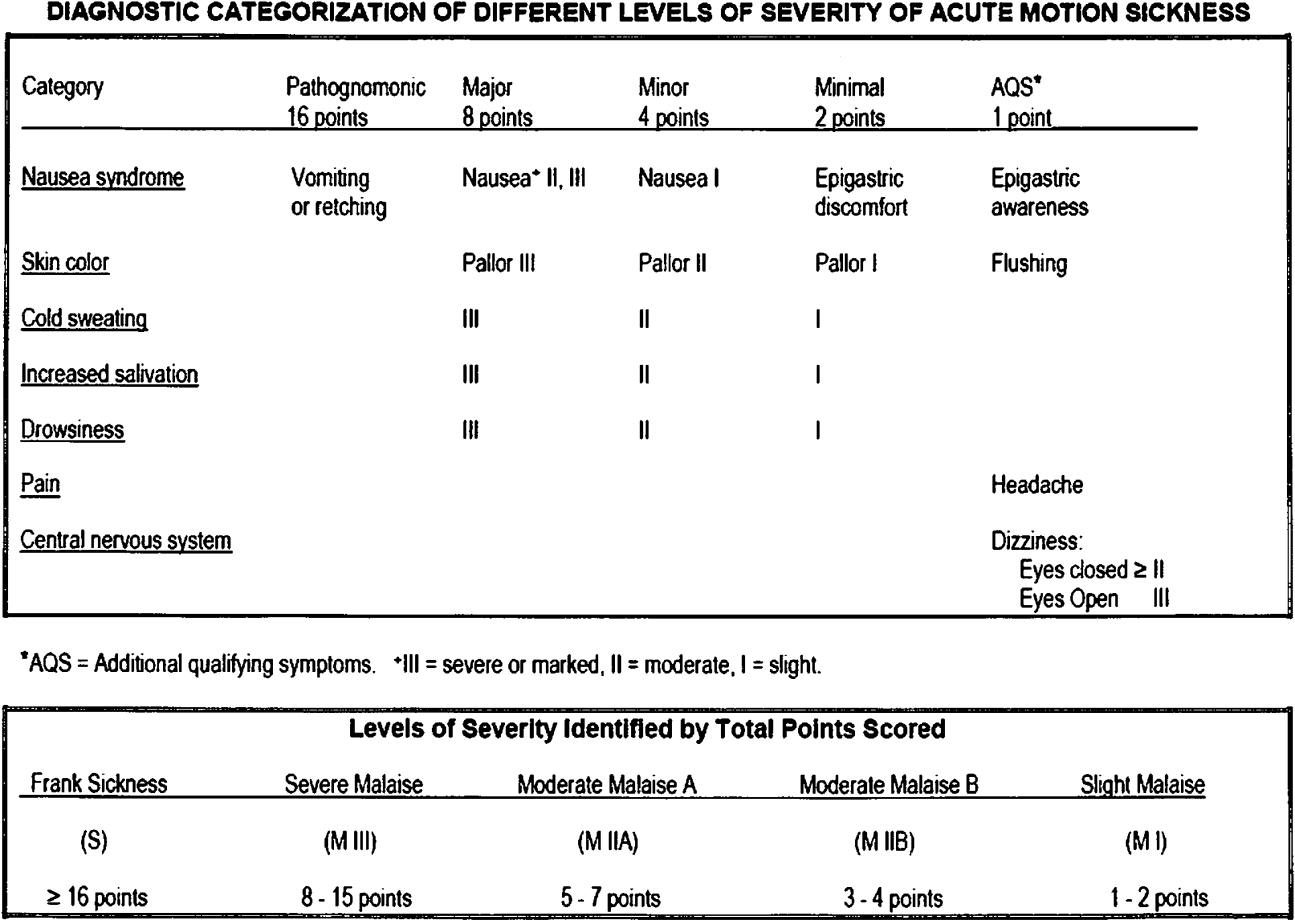
Cardinal signs and symptoms of motion sickness and criteria for grading motion sickness severity (Graybiel et al. 1968b)

Nausea and vomiting typically come to mind when people think of motion sickness. However, motion sickness comprises a much broader syndrome. Figure 1 shows a commonly used scale for identifying and rating symptoms of motion sickness. It includes a wide range of signs and symptoms including cold sweating, pallor of varying degrees, increases in salivation, drowsiness, headache, and even severe pain, as well as nausea and vomiting (Graybiel et al. 1968b). Other assessment scales rate sickness during exposure to visual or virtual stimulation and various forms of transport (Gianaros et al. 2001; Golding 1998; Golding and Gresty 2013; Kennedy et al. 1992a, b, Muth et al. 1996, Paillard et al. 2013). One facet of motion sickness that often is not recognized is the sopite syndrome (Graybiel and Knepton 1976, Lawson and Mead 1998; Matsangas and McCauley 2014a). It refers to the profound drowsiness and persistent fatigue that can follow brief exposures to highly provocative stimulation or prolonged exposures to low-intensity motion stimulation. Yawning has recently been shown to be a potential behavioral marker for onset of the sopite syndrome (Matsangas and McCauley 2014b). The sopite syndrome can persist for hours or even days and when exposure is prolonged even longer. It is characterized by boredom, apathy, failure of initiative, increased irritability, and even changes in personality. It may be one of the only syndromes that persist when nausea is not elicited or has abated.

Perhaps surprisingly, the drowsiness associated with the sopite syndrome has not been linked in laboratory studies to a decrease in sleep latency onset (Leslie et al. 1997). A recent study has shown that sleep deprivation increases susceptibility to motion sickness and impairs performance on many tasks, including the perceptual discrimination test (Kaplan et al. 2014). During severe motion sickness, although many manual performance and cognitive tasks are substantially impaired (Gresty et al. 2008; Gresty and Golding 2009), simple detection tasks such as the perceptual vigilance task (PVT) seem relatively unaffected (Kaplan et al. 2014).

## Much motion sickness goes unrecognized

This is especially likely in recreational situations and under operational conditions in the military where neither the exposure conditions nor the individual’s activity is tightly controlled. Symptoms that are actually characteristic of motion sickness may be interpreted as due to fatigue or just boredom when in fact they are being elicited by exposure to motion (Bronstein et al. 2013; Guingard and McCauley 1990; Lawther and Griffin 1986; Kennedy 1975). The point is that unless nausea and vomiting are elicited, decrements in performance may not even be recognized as being indicative of motion sickness (Lackner 1984). By contrast, under laboratory conditions, it is usually easy to recognize the onset of motion sickness because exposure conditions are carefully controlled, subjects are briefed with respect to the signs and symptoms, and stimulation intensity is generally high because the goal typically is to elicit sickness in a relatively brief period of time. Laboratory studies also involve trained observers who often can recognize the development of motion sickness in a subject prior to that individual even being aware that something untoward is happening.

Early investigators of motion sickness thought that there were two types of people: those who responded primarily to provocative stimulation with head symptoms such as headache and drowsiness, and gut responders who primarily experienced nausea and vomiting (Reason and Brand 1975). Now we know that an individual’s response depends on the relative provocativeness of the stimulation, his or her relative susceptibility, and prior experience (Bos et al. 2005; Bronstein et al. 2013; Golding 1998, 2006; Golding and Gresty 2013; Golding and Stott 1997; O’Hanlon and McCauley 1974; Paillard et al. 2013). One important fact to recognize about motion sickness is that not all sickness is created equal (Lackner and Graybiel 1986c). For example, not all situations in which vomiting is elicited are equally discomforting for different individuals. Some people after vomiting feel total relief for a period of time. Others may have a much higher threshold for the induction of vomiting so that they are much more nauseated and disabled prior to vomiting, and then vomiting gives some relief, but only partial relief, and they still remain disabled (Graybiel and Lackner 1987). Some are unable to vomit even though the level of nausea they are experiencing is such that they are desperately eager to vomit. They may be more incapacitated than people who do vomit (Graybiel and Lackner 1980). Some subjects are progressively sensitized by repeated exposures to the same motion and become sick sooner but, the majority, show progressive adaptation over time and diminution of sickness (Graybiel and Lackner 1983).

Some individuals experience great anxiety as symptoms of motion sickness develop or even before by virtue of prior exposure or impending exposure to a provocative motion environment. (Jacob et al. 1993, 1995; Money 1970; Money et al. 1996; Yardley et al. 1994). This interrelationship has been recognized since very early times. Balaban and Jacob (2001) have provided a comprehensive review covering from the very earliest writings up to 2000.

## The rate of decay of symptoms is a key factor, influencing susceptibility and performance

Decay of symptoms varies enormously across individuals. Some people on receiving provocative stimulation will show a very brief response, and others will maintain discomfort for a prolonged period. In our own studies, we have found that three key factors affect sickness development: sensitivity to stimulation, the rate of adaptation to stimulation (adaptation constant), and the time constant of decay of elicited symptoms (Ventura et al. 2014). We have found that the range of sensitivity in the general population varies about 10–1, and the adaptation constant also ranges from 10 to 1. By contrast, the decay time constant varies by 100–1. The import of these values is that susceptibility to motion sickness in the general population varies by about 10,000–1, a vast range.

The particular values of these three factors for a given individual can allow predictions of performance in different exposure conditions. For example, a person with high sensitivity to provocative motion but with a short decay constant and high rate of adaptation can—depending on the characteristics of a particular environment—experience less sickness and performance decrement than an individual with moderate sensitivity, but a long decay time constant, and a low rate of adaptability. The first individual will not show an integration or progression of symptoms with continued exposure, but will achieve adaptation quickly. The second will become progressively more motion sick because of the additive effects of continued exposure and failure to adapt.

Attempts to understand why there are such huge variations in sensitivity to motion stimulation and in decay rates have focused on factors such as estimates of sound loudness and the decay rates of the visual spiral aftereffect (Reason 1968) and asymmetries in ocular counter-rolling (Diamond and Markham 1991). Enhanced perception of sound loudness and persistence of visual spiral aftereffects were thought to reflect heightened sensitivity and persistence to sensory stimulation that might correlate with increased sensitivity to vestibular stimulation and hence susceptibility to motion sickness. Golding (2006) presents a critical review and analysis of attempts to relate susceptibility to a wide range of physical, physiological, and psychological factors. He emphasizes that, while there are many hypotheses and correlations, firm conclusions are lacking and broader knowledge of genetic factors will also be essential to a comprehensive understanding.

## Who is at risk for motion sickness?

Experimental studies have shown that virtually anyone with normal vestibular function when exposed to provocative physical body motion, disruption of vestibulo-ocular reflexes, or optokinetic stimulation can to some extent be made motion sick. Graybiel (1970) found that blind individuals are as susceptible to motion sickness when exposed to provocative physical motion (Coriolis cross-coupling stimulation, see below) as sighted individuals who have their eyes closed, and their range of susceptibility tends to be comparable. Congenitally blind subjects lack vestibulo-ocular reflexes, such reflexes are present but abnormal in individuals with acquired blindness (Kompf and Piper 1987; Leigh and Zee 1980; Sherman and Keller 1986). The individuals tested by Graybiel varied over a broad range from congenital to late acquired blindness.

The only individuals not susceptible to motion sickness under virtually every condition so far explored are those with total loss of labyrinthine function (Cheung et al. 1991; Kellogg et al. 1965; Money and Cheung 1983; Dai et al. 2007; Johnson et al. 1999; Kennedy et al. 1968; Money 1990; Money et al. 1996). Such vestibular loss subjects also tend to be immune to the action of emetic drugs (Money and Cheung 1983). Normal individuals are not always equally susceptible to all forms of motion stimulation; some may be more susceptible to full field visual stimulation than vertical or horizontal oscillation of the body. Generally, there is a correlation of about .6–.8 between individual susceptibility in one motion environment and susceptibility in another (Golding 2006, Miller and Graybiel 1972).

The introduction of head-mounted displays and smart phones with sophisticated graphics to create virtual environments has led to great increases in visually induced motion sickness, including nausea and vomiting (DiZio and Lackner 2000; Lackner and DiZio 2003; McCauley and Sharkey 1992; Stanney et al. 1998; Hettinger and Riccio 1992; Kennedy et al. 1992a, b, 1993, 2010). In fact, nearly any situation that involves suppression of vestibulo-ocular reflexes is potentially provocative. For example, sea sickness and car sickness are triggered by the motion of the vehicle. When a person is reading or looking at something that is stable within the vehicle, it is necessary to suppress the vestibulo-ocular reflexes triggered by the vehicle’s motion. In virtual environments that involve head tracking to update the visual scene, there are typically lags so that after the head begins moving, the visual array may not be updated until 60 or more msec later. Consequently, when the user moves eyes and head to focus on a peripheral area of the visual array, there will be a delayed visual sweep of the scene opposite the head movement, which provides optokinetic stimulation tending to drive the eyes off the desired fixation position. The user has to suppress this optokinetic reflex, and this can be extraordinarily provocative with a wide field of view display and time lags greater than about 60/ms. Such delays lead not only to motion sickness, but also to an increase in duration of head movements and postural instability when the person is standing (DiZio and Lackner 1997, 2000, 2002). Suppression of vestibulo-ocular reflexes is even evocative of motion sickness when the individual’s eyes in darkness. During exposure to angular acceleration, voluntary deviation of the eyes in the direction of the slow phase component of the reflexive eye movements is provocative and also increases the body displacement experienced (Evanoff and Lackner 1986; Quarck et al. 2009).

The introduction of smart phones and tablets with sophisticated graphics has also led to frequent reports of symptoms characteristic of motion sickness. Stoffregen et al. (2014) have described the important role of head and torso movements in eliciting symptoms.

## Any situation that requires altered control of the head and body is potentially provocative

For example, on shipboard, passengers have to adopt a different way of standing and to anticipate the motion of the ship, which involves an altered pattern of neuromuscular activation to achieve desired upright stance, let alone to move about. Similarly, in the weightless conditions of space flight, the whole manner in which body orientation is controlled is altered. This changed control of the body and the alterations in vestibular function (unloading of the otolith organs) occurring in weightless conditions are extremely provocative during the first several days of space flight (Lackner and DiZio 1989, 2006; Lackner et al. 1991; Thornton and Bonato 2013).

On Earth, it is profoundly provocative to make pitch or roll head movements while rotating. Such head movements lead to a bizarre pattern of stimulation of the semicircular canals (Guedry and Graybiel 1962; Miller and Graybiel 1970a, b) and the generation of a Coriolis force on the head that will tend to deflect it from its intended path. Under normal non-rotating conditions when a head movement is made, the semicircular canals in the plane of head motion will be stimulated by the acceleration of the head. The endolymph in the canals will lag and displace the cupulae, but then as the head decelerates, the cupulae will be restored back to their rest position. The acceleration and deceleration of the head are typically completed in much less than one second. The neural output of the semicircular canals is then actually proportional to head velocity because the inertial and mechanical properties of the cupula-endolymph system essentially perform a mechanical integration. This head velocity signal is used to control compensatory eye movements that normally are appropriate for the situation. In addition, that signal is integrated to give an indication of the angular displacement of the head relative to space (Wilson and Melvill Jones 1979; Baloh and Honorubia 1990; Cohen et al. 1977, 1981).

**Fig. 2:**
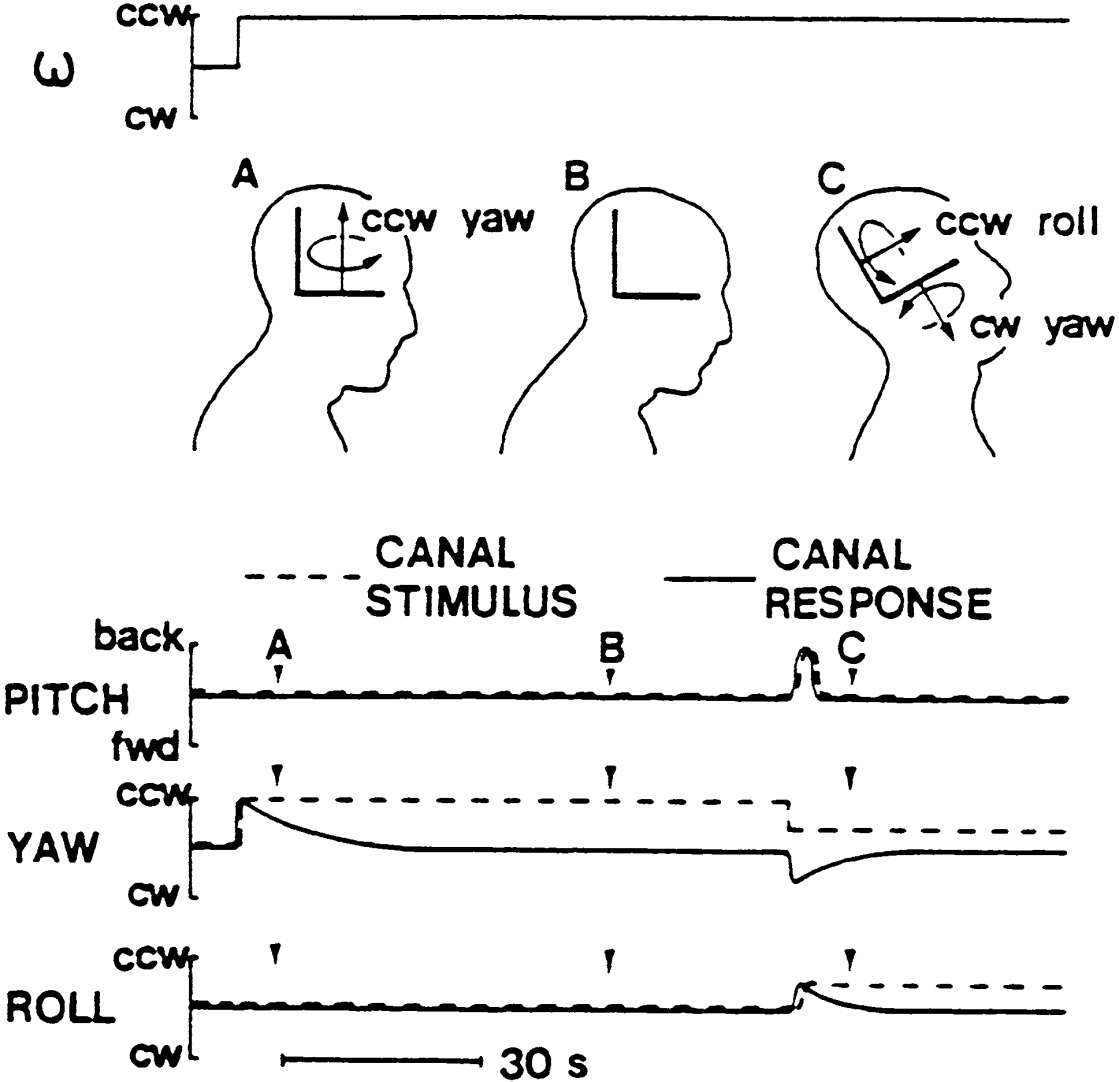
Illustration of Coriolis cross-coupling stimulation

The left side of Fig. 2, which illustrates only one member of each of the three pairs of bilaterally symmetric canals, shows that during exposure to a step change in horizontal angular velocity, the “yaw canal” cupula will be deflected and only gradually return to its rest position(Guedry and Benson 1978). Consequently, body rotation is felt and steadily decreases in magnitude until at point B the subject feels stationary again. But, when a pitch back head movement is made at C, the yaw canal is tilted out of the plane of rotation and loses angular momentum so that it is stimulated in the opposite direction of that initially. The “roll canal” is brought into the plane of rotation and receives a step change in velocity so that it also responds for a prolonged time. The “pitch canal,” by contrast, accurately signals the head movement that occurred in pitch. As a consequence of this unusual pattern of stimulation, the subject senses a complex pattern of body rotation and displacement, which persists until the canal signals decay back to baseline. This “Coriolis cross-coupling” stimulation is extremely disorienting and nauseogenic, and most people can only make a small number of head movements before becoming severely motion sick (Miller and Graybiel 1970a, b).

## Studies of space motion sickness enhance our understanding of terrestrial motion sickness

**Fig. 3:**
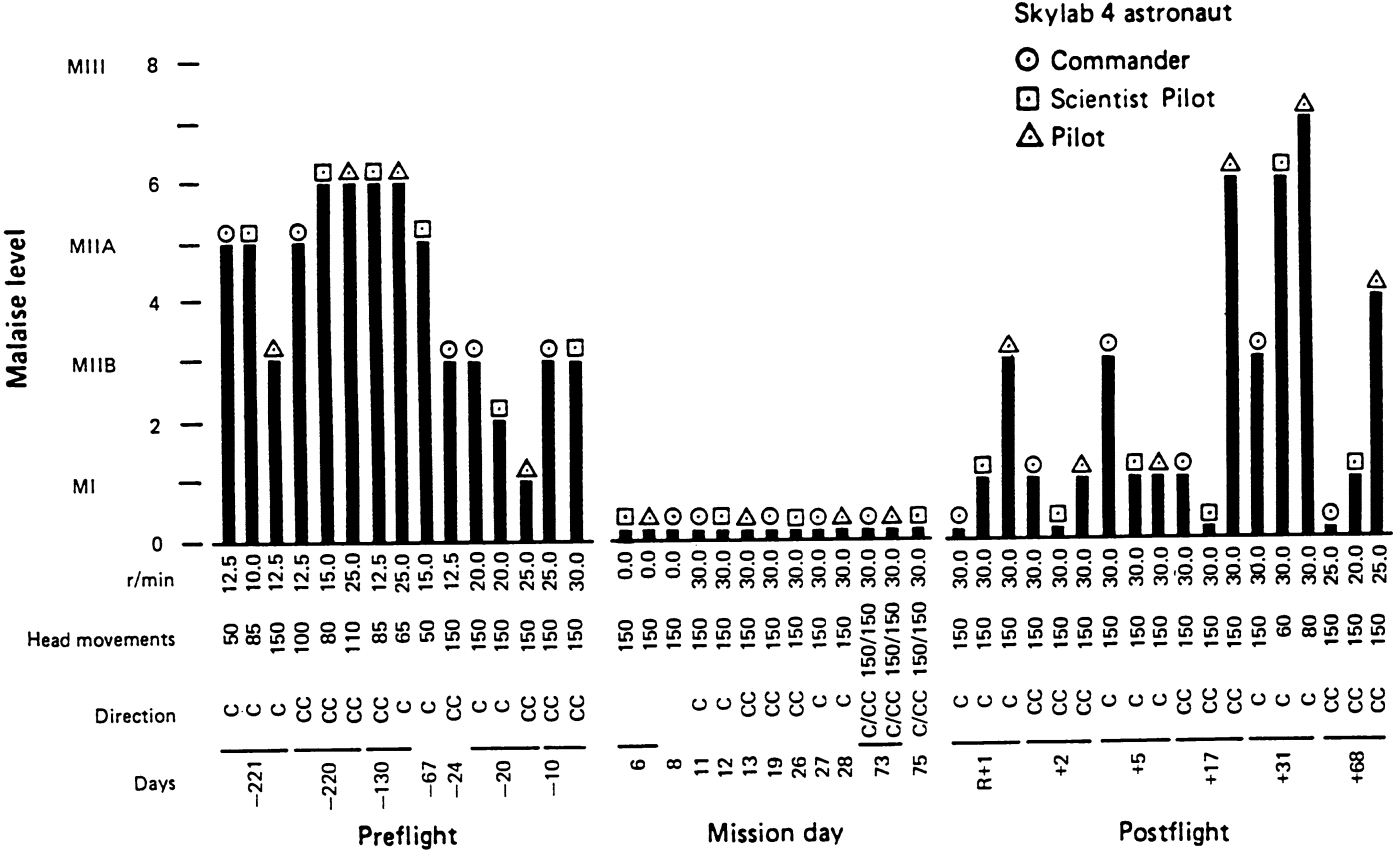
Results of the Skylab M-131 experiment for the three Skylab 4 astronauts. The *columns* represent the severity of motion sickness on the Graybiel scale (see Fig. 1). The *rows* of numbers at the *bottom of the figure* represent the angular velocity of body rotation in rpm, number of head movements made, rotation direction, and flight day

In systematic studies of responses to Coriolis cross-coupling stimulation conducted in space flight, the Skylab 131 experiment, a startling result was obtained (Graybiel et al. 1975, 1977; Miller and Graybiel 1973). The participating astronauts had been tested preflight for their sensitivity to Coriolis cross-coupling stimulation and had been highly susceptible. Nine astronauts were tested over the course of the three manned Skylab missions. When tested in-flight each was totally insusceptible. Coriolis cross-coupling stimulation no longer “tumbled their gyros.” Figure 3 shows the results of the Skylab 4 flight (Skylab 1 was an unmanned mission.). These findings presented a quandary. How could astronauts be susceptible preflight, but not during flight? A semicircular canal in terms of its mechanical properties should be gravity independent because the densities of the cupula and of the endolymph are each virtually one. Consequently, there is no gravity couple acting on the endolymph and cupula of a semicircular canal (Wilson and Melvill Jones 1979). Neither gravity nor gravito-inertial force level should directly influence canal neural output.

**Fig. 4:**
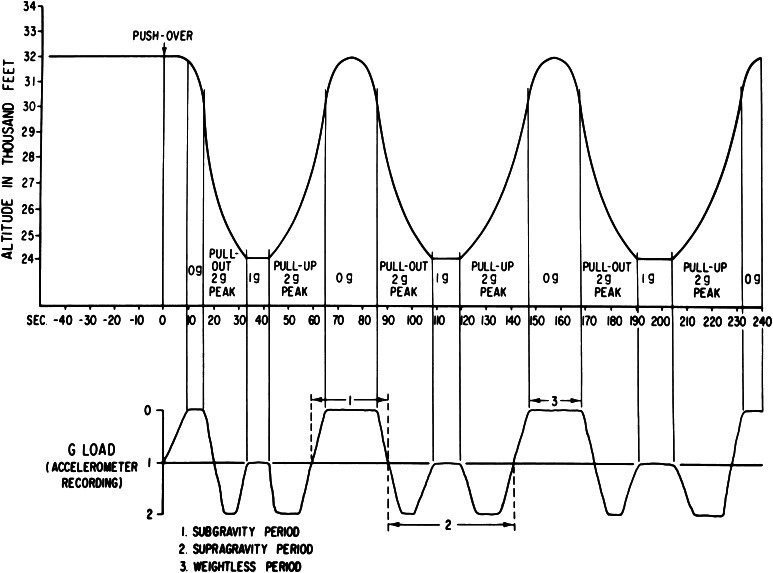
Schematic flight profile of aircraft performing parabolic maneuvers to generate alternating period of free fall and increased gravito-inertial force

Studies were conducted in parabolic flight to determine the basis for the Skylab findings. Blindfolded subjects rotating at constant velocity made head movements while exposed to weightlessness (0 g), to straight and level flight, and to 2 g background force levels, where *g* = 9.8 m/s^2^, the acceleration of Earth gravity (see Fig. 4). The results were unequivocal (Lackner and Graybiel 1984a, 1986a). Immediately on transition into 0 g, head movements during rotation were less provocative and less nauseogenic than head movements made in straight and level flight. By contrast, head movements in 2 g were much more provocative than in level flight, and most subjects could only make a few before becoming nauseated to the point of vomiting. Moreover, being in weightlessness (“0 g”) eliminated the disorienting effects of the head movements; the head movements felt nearly normal and were no more provocative than a head movement made in 0 g while not rotating (Lackner and Graybiel 1983, 1984b, 1985, 1986b).

Additional studies evaluated whether the velocity integration normally associated with canal stimulation to give a sense of angular spatial displacement was affected in 0 g. It had already been found that the nystagmus that results from Coriolis cross-coupling stimulation was greatly suppressed in 0 g relative to 2 g (DiZio et al. 1987a), as was the nystagmus resulting from sudden-stop stimulation from constant velocity rotation (DiZio et al. 1987b). These findings raised the possibility that the central integration of the canal velocity signal was disrupted. To test this hypothesis, subjects were placed supine in a cradle-like device and used a joystick to indicate the amplitude of the angles through which they were turned when they were exposed to rotary angular accelerations an order of magnitude greater than threshold detection levels in 0, 1, and 2 g (Lackner and DiZio 2009).

**Fig. 5:**
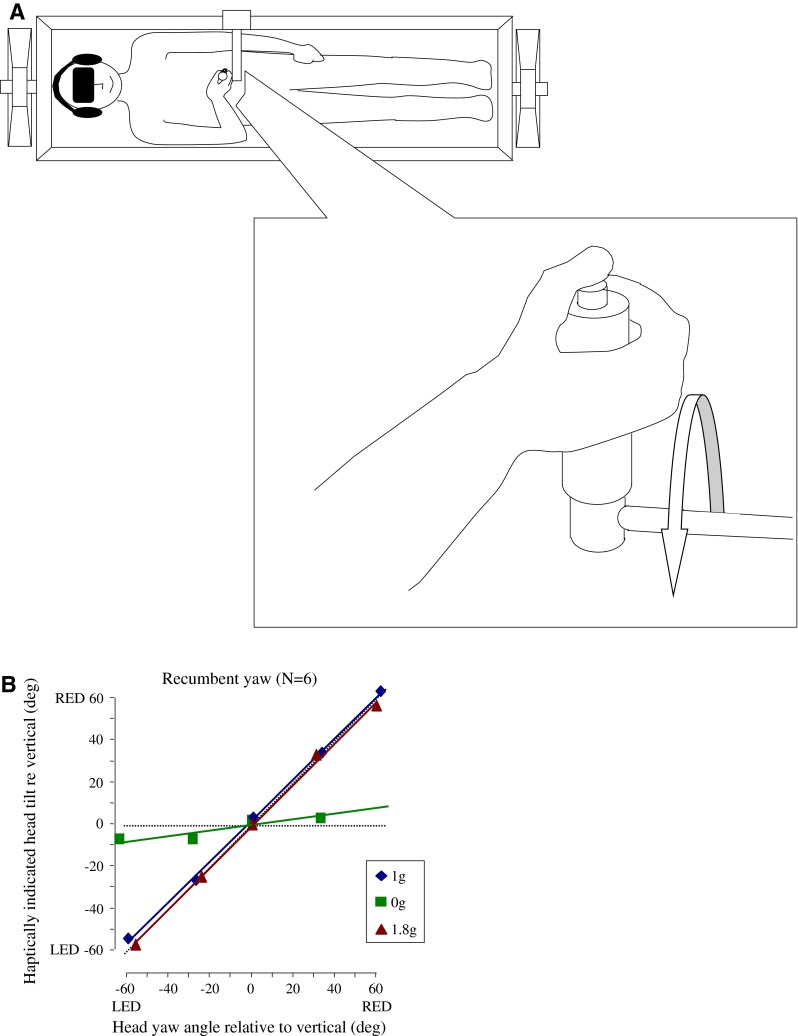
Angular displacement perception as a function of gravito-inertial force level for recumbent yaw rotation. *LED* left ear down, *RED* right ear down

Figure 5 shows that in 1 g, the blindfolded subjects were accurate in indicating their angular displacement. By contrast, in 0 g, they made a slight initial joystick movement in the direction of the turn, but then kept it aligned with their body axis because they did not feel any spatial displacement, just a slight initial tug in the direction they had actually been turned. These results mean that in a weightless environment, the signals from the semicircular canals are not being integrated by the central nervous system to give rise to a sense of body spatial displacement. It also explains why the Skylab astronauts were not susceptible to Coriolis cross-coupling stimulation in-flight. The signals that give rise to spatial displacement and lead to disorientation were not generated. It also explains why astronauts sometimes lose track of their orientation in space vehicles and may fail to recognize their spatial location when they make body turns (Lackner and DiZio 2000a).

During the Skylab M131 experiment, the astronauts were first tested on or after mission day 6. Coriolis cross-coupling stimulation was thought to be so provocative that in-flight testing was delayed for many days lest the astronauts become so motion sick that it would disrupt their other activities for days. During the initial days of the missions, the astronauts, however, did become motion sick simply by virtue of making head and body movements. Being in a weightless environment alters the sensory motor control of the head as an inertial mass, and we now know that such alterations are provocative per se (Lackner and DiZio 1989, 2006; Lackner and Graybiel 1980, 1986b; Oman 1987; Oman et al. 1986, 1990; Thornton and Bonato 2013). This altered control and need to recalibrate are a major factor in space motion sickness and terrestrial motion sickness experienced in vehicles as well.

The crucial point is that motor control is normally dynamically tuned and calibrated to the 1 g background force of earth. Whenever deviations or variations from this force level occur, motion sickness may result because of the disruption of vestibulo-ocular, optokinetic, and collic reflexes that need to be retuned. Motion sickness can persist until a new pattern of control of the head and eyes has been attained, and accommodation is made to the remapped relation between voluntary control of the head and body and the vestibular activity evoked (DiZio and Lackner 1997; Lackner and DiZio 1992, 2000a, b, 2006).

## Physiological mechanisms

The physiological mechanisms underlying the elicitation and expression of motion sickness are complex and still not fully known (Yates et al. 1998). Miller and colleagues have identified the neural circuits controlling the respiratory and abdominal muscles during nausea and vomiting, and which brain areas are critical (Miller and Grelot 1996; Miller et al. 1990, 1994; Miller and Wilson 1983). Balaban has described the complex interrelationships between mechanisms subserving balance control and those related to anxiety and fear responses (Balaban 1996, 2002; Balaban and Thayer 2001). These pathways likely underlie the evocation of anxiety and dread some individuals experience as they begin to become motion sick. The relationship may actually be bidirectional, with anxiety and fear also enhancing the severity of motion sickness. Recently, the brain areas active during the development of nausea have been identified in imaging studies (Napadow et al. 2013; Sugiyama et al. 2011; Suzuki et al. 2012; Yates 1996a, b; Yates et al. 1995a, b; Yates and Bronstein 2005; Yates and Miller 2009).

Major recent progress has been made by Yates and his collaborators who have delineated the brain stem regions implicated in the elicitation of nausea and the control of the muscles involved in emesis. Yates et al. (2014) have provided a comprehensive description of the pathways involved in the evocation of nausea and vomiting. The pattern generator circuits involved in the actual act of vomiting have now been identified. Brain stem areas including the nucleus of the solitary tract (NTS), the dorsolateral reticular formation of the caudal medulla (lateral tegmental field, LTF), and the parabrachial nucleus (PB) together integrate signals that lead to nausea and vomiting. The detailed innervation and coordination of the diaphragm and abdominal muscles to evoke vomiting is now understood. During quiet breathing, their activity is in anti-phase, but during vomiting (and a range of activities involving postural stabilization), their activity is synchronized. Both PB and LTF responses are influenced by visceral afferents that also alter the responses to labyrinthine stimulation. NTS is the terminus of many visceral afferents and also receives efferent projections from the area postrema, which was once thought to be the primary “vomiting center.” NTS is now known to relay signals to the emesis pattern generator circuit. Neurons in the vestibular cerebellum, including the fastigial nucleus (FN), are also influenced by visceral afferents. These regions may be implicated in triggering motion sickness, and Brooks and Cullen (2013) have recently shown that FN is very much involved in movement control and its adaptive updating.

 In an elegant series of studies, Yates and his colleagues have shown the important role of the vestibular system in the regulation of respiration, heart rate, and compensations for changes in body orientation re gravity. For example, stimulation of cervical roots C2 and C3 alone affects the hypoglossus (tongue protrusion), but not respiration, whereas changes in head and body orientation elicit compensatory changes in respiration, as well as tongue protrusion (Bolton et al. 1998). This dichotomy ensures that changes in head orientation relative to a stationary torso are not conflated with head and torso changes re space (Moy et al. 2012).

Jian et al. (2002, 2005) have found that both somatic limb afferent stimulation and visceral afferent stimulation affect responses of cells in the vestibular nuclei during vertical rotation of the body in cats. In labyrinthectomized cats, even a larger percentage of the cells are affected. As the authors point out, the inputs of non-labyrinthine origin may be associated with and triggered by particular active behaviors. Yates et al. (2002) have shown that passive changes in body orientation with respect to gravity affect respiration. During shifts from supine to upright body orientation, the “length” of the diaphragm changes. The change in vestibular activity associated with going from a supine to erect orientation produces increases in diaphragm and abdominal muscle activity, which aid venous return. Labyrinthectomized animals lose this response, but recover it over time based on remaining signals about body orientation. Respiratory pump muscle activity is also affected by cerebellar regions receiving vestibular inputs—these influences can be excitatory or inhibitory, and it is uncertain whether they are engaged during voluntary movements.

Rice et al. (2010) have shown using rabies tracing techniques that cells in the inferior and lateral vestibular nuclei and in the medial pontomedullary reticular formation (MRF) influence diaphragm activity and project to the lumbar spinal cord. This pattern of connectivity suggests an influence on the integrative coordination of the diaphragm in situations involving voluntary (and perhaps passive) movements of the body. Yates and his collaborators have shown that there is a strong influence of visceral stimulation on the vestibular system (Arshian et al. 2013). Intragastric delivery of copper sulfate activates visceral afferents and can evoke both nausea and vomiting. These afferents also modulate, both up and down, the activity level of neurons in the caudal vestibular nucleus during vertical oscillation. Other areas of the vestibular system are even more affected by copper sulfate ingestions, with neuronal discharge levels increased. Other experiments by the Yates group have shown powerful influences of vestibular activity on respiration and heart rate (Yates et al. 2000). These findings together show how vestibular activity associated with body motion helps regulate heart rate and respiration.

The Yates studies together provide a long-needed basis for understanding why exposure to passive body motion may be provocative and why it is frequency dependent and dependent on body orientation re the direction of gravity (Anker et al. 2006; Rossiter et al. 1996; Wiker et al. 1979). For vertical oscillation, the most nauseogenic frequency is circa .2 Hz (O’Hanlon and McCauley 1974). This value is below that for voluntary body movements involving locomotion and head or torso orienting movements. However, it is within the frequency of vertical motion experienced when riding a camel, which is notoriously provocative. Thus, there is rare human experience with vertical body motion at this frequency. Nevertheless, low frequency vertical oscillation will cause inertial lag of the viscera and excite a broad range of visceral mechano-receptors. Recently, it has been shown that gut vagal afferents also influence anxiety and learned fear (Klarer et al. 2014).

## Theories of motion sickness

Many theories of motion sickness have been proposed over the years. The *evolutionary theory* holds that motion sickness is essentially a response to poisoning (Money 1990; Treisman 1977). The notion is that when a noxious substance is ingested (e.g., rotting flesh) if nausea and vomiting result, inactivity will be induced and symptoms will be attenuated because of reduced levels of toxins circulated in the blood stream. Decreased activity enhances the possibility of recovery. This theory has empirical support. It is well known that people without functioning labyrinths are virtually immune to emetic drugs such as ipecac. Dogs have long been used in animal studies of motion sickness because their susceptibility patterns and emetic responses are similar to those of humans (Wang and Chinn 1956). The reactions of dogs to emetic drugs is usually greatly reduced or absent after they have been labyrinthectomized compared with their baseline responses (Money and Cheung 1983; Money et al. 1996).

A skeptic might argue that from an evolutionary standpoint susceptibility to motion sickness is actually a disadvantage. Evolutionary biologists long have recognized the need for balancing energy demands with the extent of body fatness and invoke the notion of set points for regulating “fatness.” A complex set of hormones and receptors in the alimentary tract responds to digested foods and affects appetite drive. They also have influences on the nucleus of the solitary tract (NTS) as well as the arcuate nucleus of the hypothalamus, which can affect set points (da Silva et al. 2014; do Carmo et al. 2013; Sohn et al. 2013; Speakman 2014). The lower set point of body fatness—for regulating food intake—protects against death by starvation, whereas the upper set point limits body fatness, which lowers the risk of death by predation (Gosler et al. 2002, Higginson et al. 2012). In the evolutionary history of man, Australopithecus was heavily preyed on by dinofelis, a type of saber-toothed cat with especially strong forelimbs for grasping prey. It hunted paranthropus and homo habilis as shown by skulls found with the typical twin punctures delivered by saber canine teeth. Only with the acquisition of fire and tool use and weapons did death from predation become rare (Eller et al. 2009; Harding et al. 1997; Speakman 2014). Thus, from an evolutionary standpoint greatly limiting the capability to move by inducing nausea and vomiting would have decreased survival likelihood in the presence of prey. This alternative interpretation of the evolutionary theory would identify susceptibility to motion sickness as a pruning factor rather than a safety mechanism!

The *ecological theory of motion sickness* is based on the hypothesis that motion sickness is caused by postural instability, a loss of postural control (Riccio and Stoffregen 1991). It is a corollary of the ecological theory of orientation that holds that perception of the upright is determined by the direction of dynamic balance (Stoffregen and Riccio 1988). The concept is that as postural instability increases, motion sickness will develop (Smart et al. 1998; Stoffregen and Smart 1998). Support for the ecological theory of orientation was derived from experiments in which blindfolded subjects used a joystick to set themselves to the “upright” by controlling a device programmed to mimic inverted pendulum behavior. The direction of balance of the device could be offset from the gravitational vertical by as much as ±20°. With an offset direction of balance, the device’s stability point does not correspond to the gravitational vertical. The experimental data indicated that subjects’ settings of themselves to the upright were influenced by the apparatuses’ direction of balance. These data seemed to confirm that dynamic balance influences the perceived upright.

Recently, the ecological theory of orientation was re-evaluated in experiments in which blindfolded subjects controlled a device with inverted pendulum dynamics whose direction of balance could be offset as much as ±30°. Using a joystick, they set the device to four different instructed orientations: (1) the direction of gravity, (2) the upright, (3) the direction of least oscillation, and (4) the direction of balance. They also pressed the joystick trigger each time they were at the desired orientation (Panic et al. 2013a, b, 2014a, b). The attained settings of the apparatus were not different for the gravitational vertical and the upright, and corresponded to the results of Stoffregen and Riccio (1988). The joystick trigger presses, however, corresponded with the gravitational upright, not the direction of balance. By contrast, for setting the apparatus to the direction of balance and pressing the trigger, the attained and indicated settings were displaced in the direction of gravity. Subjects passively exposed to motion profiles of the device, which had been recorded when other subjects were actively controlling the device, were also accurate in indicating the direction of gravity with a trigger press. These results are in direct contradiction to the ecological theory of orientation and emphasize the importance of gravity rather than dynamic balance. Subjects sitting outside the device controlling it visually performed similar to subjects controlling it while in it.

Studies of the relationship between postural instability and onset of motion sickness also show little support for the ecological theory of motion sickness (Owen et al. 1998; Warwick-Evans and Beaumont 1991; Warwick-Evans et al. 1998). It is likely that the conditions in which postural instability precedes “motion sickness” are actually conditions in which postural hypotension is being elicited, hence symptoms such as “can’t see straight, feeling dizzy,” “hard to focus, everything is gray,” and occasionally nausea (Smart et al. 1998, 2002; Stoffregen et al. 2010; Villard et al. 2008). Typically, the ecological experiments involve prolonged visual fixation during passive upright stance at target distances that can range from .3 to 2 m, or oscillation of the visual scene or surroundings. In similar conditions, we have had several episodes of full syncope. Subjects who are most affected tend to have low blood pressure. Controlled studies of balance and onset of motion sickness for exposure to virtual ship motion have shown the trend that motion sickness symptoms develop and then postural performance degrades (DiZio and Lackner 1997, 2000, 2002).

It is generally believed that the driver of a vehicle almost never becomes motion sick, whereas passengers may—and there is evidence to support this perspective (Rolnick and Lubow 1991). This viewpoint is consistent with a classic body of research supporting the view that active movement is essential in order to adapt to sensory rearrangement—e.g., prism spectacles that displace the visual array or to develop normal sensory motor coordination (Held and Hein 1958, 1963). However, these studies failed to control for the role of attention. When active and passive exposure conditions are equated in terms of attention demands—what the subjects are instructed to do—significant differences are typically absent or minimal Lackner 1981, Mather and Lackner 1981). Indeed, adaptation is minimal or absent when an individual makes movements that are perturbed and the instruction is to simply repeat the same movement; but if the instruction is to reach and attain a target goal, then adaptation is rapidly achieved (Kurtzer et al. 2003).

In “yoked paradigms,” such as employed by Rolnick and Lubow (1991) where one individual initiates a movement and controls its parameters and the passive participant undergoes the same motion, it is important to have the subjects matched in terms of attentional demands and expected motion whether generated actively or passively. We have created a situation in which two subjects are seated side by side on a rotating device. One subject—the active subject rotates a handle mounted on a rheostat that activates the motor controlling the device. The passive subject’s hand rests on a handle attached by a linkage to the active subjects control handle and passively receives indication of the physical motion forthcoming some milliseconds later. No difference in motion sickness susceptibility is present between active and passive subjects indicating being able to anticipate, actively or passively, impending motion is key. This ability to anticipate likely represents the ability of the cerebellum to predictively model the motion environment, is recently described by Bhanpuri et al. 2013.

The *sensory conflict theory of motion sickness* proposed by Reason (1969, 1970, 1978; Reason and Brand 1975) was developed into a quantitative model by Oman (1982, 1990, 1998). It is the most widely accepted theory of motion sickness. Nearly all situations that elicit motion sickness involve some form of sensory motor conflict (Bles et al. 1998). Recent work by Cullen and colleagues on the cerebellar and vestibular mechanisms related to the control and appreciation of head and body movements has shown the important relationship between corollary discharge signals and reafferent signals associated with the resulting movement of the head or body. Any discrepancy between the expected and reafferent signals represents a sensory conflict that potentially could be provocative (Brooks and Cullen 2009, 2013; Cullen 2012; Cullen et al. 2009; Saab and Willis 2001; Serra et al. 1998).

Sensory conflict theories typically relate voluntary commands to the musculature (corollary discharge signals) to expected patterns of afferent signals (reafference) from vision, touch, hearing, proprioception and vestibular activity. However, it is important to realize that whenever arm movements, or virtually any whole body activity is executed the soft tissues of the body are also affected—e.g., lungs, kidneys, viscera, bladder, heart. For example, during activities in which surges in abdominal muscle activity and diaphragm activity increase pressure on the bladder and colon, anticipatory innervations of pelvic floor sphincter muscles are necessary to prevent leakage of urine and feces (Campbell et al. 2012; Hodges et al. 2007). Respiratory rhythms have to be appropriately entrained to locomotion to ensure efficient coordination of skeletal muscle activity and the “bouncing viscera” during running. Somatic afferent stimulation contributes to this entrainment, which can be prevented by blockade of the PB nucleus (cf Daley and Usherwood 2010; Porterfield 1985; Potts et al. 2005). Levinthal and Strick (2012) have shown that multiple motor and non-motor areas of cortex directly influence kidney function, including M1, M2, S1 and the insula. M1 and M2 have especially important contributions that arise from their respective trunk representation areas in motor cortex. These pathways provide a source of commands to somatic musculature as well as for sympathetic control of the kidneys.

Lovejoy (1988) has summarized some of the skeletal and muscular adaptations associated with visceral control during the transition to upright walking in Australopithecus (“Lucy”). Others have highlighted the way in which motion of the viscera is controlled by abdominal, diaphragm, and chest muscle activity during locomotion (Simons 1999). For example, during brachiation, a valsalva maneuver (forced expiration with closed air passages) is executed to rigidify the rib cage (Napier 1993; Wilson 1998). This allows the arms to exert against a stable base the substantial forces necessary to propel the body from branch to branch. The key point is that whenever a voluntary movement of the body is made many other motor compensations are simultaneously taking place outside of conscious awareness that are essential for the successful completion of the movement. These anticipatory postural compensations ensure stability of postural control and balance but also generate patterns of afferent feedback from both somatic and visceral receptors. Most of these afferents do not reach conscious awareness unless something goes awry. For example, an individual with a collapsed lung who makes an inspiratory movement may feel an empty space, a cavity in the chest. Many internal signals under normal circumstances are subject to sensory inhibition as so elegantly shown in von Bekesy’s classic experiments (cf von Bekesy 1967). The point is that the nervous system precludes from consciousness many signals related to the background activity subserving specific volitional goals.

Sensory conflict theory must incorporate these ancillary signals from viscera and other internal organs when modeling the implications of exposure to conflict situations. For example, when on a moving vehicle such as a ship, getting one’s “sea legs” involves being able to coordinate whole body movements to achieve desired goals in the moving environment while also maintaining appropriate predictable stabilization of the viscera. Visceral afferents, as discussed above, affect the control of respiration and heart rate and vestibular sensitivity to motion. Cerebellar mechanisms related to the formation of internal models of motor and sensory control thus have to incorporate models of the environment to which the organism is exposed and must adapt to, e.g., predictable vehicle motion. Here the concept of allostasis and the vestibulo-cerebellar and cerebellar-cortical reciprocal mechanisms involved in the regulation of the internal and the external motor and sensory milieus figure prominently (Bastian 2011; Bhanpuri et al. 2013; Brooks and Cullen 2009, 2013; Christensen et al. 2014; Criscimagna-Hemminger et al. 2010; Cullen 2012; McEwen and Wingfield 2010; Scott 1994; Strick et al. 2009; Wingfield 2003). The systematic pioneering work of Yates and his colleagues described above has shown the key importance of visceral and vestibular and cerebellar afferent signals in relation to motor ones in the adaptive maintenance of allostasis in different environments.

What remains perplexing, however, is why some conflicts are provocative and others are not. A common laboratory technique for studying motion sickness is to have subjects seated inside a large vertically striped drum. When the drum is rotated at constant velocity, it will soon be seen as being stationary and the subject will feel constant velocity self-rotation in the direction opposite that of the actual drum motion. Most subjects will develop symptoms of motion sickness within minutes (Hu and Stern 1998; Koch 1999; Lawson 1993; Stern et al. 1993, 1985, 1987). By contrast, if the subject is walking forward on a rotary treadmill moving in the same direction and at the same rate as the surrounding drum, no motion sickness will result (Lackner and DiZio 1988). Instead, the subject will feel voluntary self-motion in relation to a stationary drum, and the actual visual stimulation will be consistent with this. In other words, there is no conflict, and sensory conflict theory predicts no sickness. However, if the direction of the rotating drum is reversed while the subject continues making forward stepping movements on the treadmill, he or she will soon experience backwards motion. Some subjects in this circumstance feel that they are voluntarily making backwards stepping movements, others feel a paradoxical sense that their forward steps push them backwards. Despite the profound sensory conflict, no sickness is experienced, but instead, there is a central remapping of the subject’s experienced activities that make them consistent with the visual flow. Surprisingly, subjects who make voluntary head movements during exposure to moving visual stimulation can prevent the induction of self-motion and prevent becoming motion sick (Lackner and Teixeira 1977).

## How can motion sickness be avoided or attenuated?

The only sure cure is to avoid exposure to provocative situations entirely, or less desirably, to be without a functioning labyrinth. However, it is possible to introduce exposure gradually and initially limit activity in the novel environment. In fact, incremental exposure, progressively increasing the intensity of stimulation over multiple exposures, is a very effective way to prevent motion sickness (Graybiel and Wood 1969; Graybiel et al. 1969; Yen-Pik Sang et al. 2005).

A long series of pioneering experiments in the Pensacola slow-rotation room showed that it is possible to desensitize individuals by having them make head movements at very low velocities of rotation, e.g., 1 rpm, and then additional head movements at progressively higher velocities. With this paradigm, it is possible to adapt people to rotational velocities of 25 rpm, and even higher, without eliciting any symptoms of motion sickness. As a consequence of this exposure, the time constant of canal velocity storage is reduced. Motion sickness sensitivity is decreased for exposure to other forms of provocative stimulation as well (Cramer et al. 1978; Graybiel and Knepton 1978a, b; Graybiel et al. 1968a, b; Reason and Graybiel 1969, 1970).

This reduction in time constant of velocity storage is the factor that accounted for the absence of sensitivity to Coriolis cross-coupling stimulation in the weightless conditions in space flight and parabolic flight. The linear acceleration sensitive otolith organs that normally signal head orientation relative to gravity are unloaded in weightless conditions. A recent model of vestibular function indicates that in this circumstance, an otolith output specifying a determinate orientation would be absent and thus predicts velocity storage would be absent in 0 g (Bortolami et al. 2006). Vestibular loss subjects are immune to motion sickness as mentioned above and, of course, lack velocity storage.

Anti-motion sickness drugs potentially can enhance the rate of adaptation by allowing progressive exposure to higher levels of stimulation without symptoms being elicited (Cohen et al. 2008; Lackner and Graybiel 1994; Levine et al. 2000). With incremental exposure, people also can develop context specific adaptations so that, for example, they can move between a rotating artificial gravity environment and a stationary environment without either sensory motor control or motion sickness problems (Graybiel and Knepton 1978a, b; Lackner and DiZio 2000a, b, 2006). Drugs such as promethazine and scopolamine provide protective benefit (Bar et al. 2009; Davis et al. 1993; Graybiel and Lackner 1987; Gordon et al. 2001; Klocker et al. 2001; Nachum et al. 2001, 2006; Simmons et al. 2010). These drugs are central nervous system depressants and induce drowsiness so that they are often used in combination with dexedrine and ephedrine, respectively. In drug studies of motion sickness, there are always large placebo effects so that it is necessary to have both placebo and non-placebo controls. Placebo effects usually are on the order of 10–40 % of drug effects (Wood and Graybiel 1968, 1969, 1970; Graybiel et al. 1976; Wood et al. 1986). Wrist acupressure bands and magnets sold to alleviate or prevent motion sickness potentially provide placebo benefits for some people. (Miller and Muth 2004). Ginger has also been touted as a remedy, but its effects are marginal (Lien et al. 2003). Autogenic feedback training has been used in conjunction with incremental exposure to increasingly provocative stimulation as a way of decreasing susceptibility to motion sickness (Cowings and Toscano 1982, 2000). Exposing subjects to visual–vestibular interactions has been shown to reduce their sensitivity to motion sickness during travel in transports such as buses (Dai et al. 2011). The procedure works by decreasing the time constant of velocity storage.

An important challenge for the future will be to try and develop drugs for preventing motion sickness that do not have undesirable side effects such as drowsiness. The sickness resulting from chemotherapy treatments involves visceral afferent activation. An important achievement will be to develop drugs that alleviate both nausea and vomiting, not just the vomiting elicited by chemotherapy (Yates et al. 2014).
